# Canadian Medical Education Journal Survey evaluations of University of British Columbia residents’ education and attitudes regarding palliative care and physician assisted death

**Published:** 2017-02-24

**Authors:** David Spicer, Sonia Paul, Tom Tang, Charlie Chen, Jocelyn Chase

**Affiliations:** 1 Internal Medicine Residency Program, University of British Columbia, British Columbia, Canada; 2 Family Medicine Residency Program, University of British Columbia, British Columbia, Canada; 3 Division of Palliative Care Medicine, University of British Columbia, British Columbia, Canada; 4 Division of Geriatric Medicine, University of British Columbia, British Columbia, Canada

## Abstract

**Background:**

Little prior research has been conducted regarding resident physicians’ opinions on the subject of Physician Assisted Death (PAD), despite past surveys ascertaining the attitudes of practicing physicians towards PAD in Canada. We solicited British Columbia residents’ opinions on the amount of education they receive about palliative care and physician assisted death, and their attitudes towards the implementation of PAD.

**Methods:**

We conducted a cross sectional, anonymous online survey with the resident physicians of British Columbia, Canada. Questions included: close-ended questions, graded Likert scale questions, and comments.

**Results:**

Among the respondents (n=299, response rate 24%), 44% received ≥5 hours of education in palliative care, 40% received between zero and four hours of education, and 16% reported zero hours. Of all respondents, 75% had received no education about PAD and the majority agreed that there should be more education about palliative care (74%) and PAD (85%). Only 35% of residents felt their program provided them with enough education to make an informed decision about PAD, yet 59% would provide a consenting patient with PAD. Half of the respondents believed PAD would ultimately be provided by palliative care physicians.

**Interpretation:**

Residents desire further education about palliative care and PAD. Training programs should consider conducting a thorough needs assessment and implementing structured education to meet this need.

## Introduction

In February 2015, the Supreme Court of Canada (SCC) ruled that the ban on physician assisted death (PAD)* was unconstitutional. This decision was based on section seven of the Canadian Charter of Rights and Freedoms,^1^ stating that it will be a legal option for “a competent adult person who (a) clearly consents to the termination of life and (b) has a grievous and irremediable medical condition (including an illness, disease or disability) that causes enduring suffering that is intolerable to the individual in the circumstances of his or her condition.”^2^ In response to the SCC ruling, the federal government passed Bill C-14 on June 17^th^, 2016,^3^ providing a legislative framework whereby PAD can be provided by medical practitioners. In turn, provincial colleges and health care institutions have been primarily responsible for the regulation, delivery, and monitoring of PAD at the local level.

Canadian physicians’ attitudes towards the legalization of PAD have been documented in surveys and position statements published by various physician societies. A Canadian Medical Association (CMA) survey in 2014 reported that 56% of respondent physicians supported legalizing PAD and 27% would participate in the provision of PAD.^4^ In 2015, a College of Family Physicians of Canada (CFPC) ePanel identified that 58% of its members agreed with the SCC ruling, and 50% would provide PAD if the appropriate checks and balances were in place.^5^ The Canadian Society of Palliative Care Physicians (CSPCP) has stated: “Many requests for physician-hastened death are indications of suffering that could be ameliorated by Palliative Care. If patients were able to receive high quality palliative care, requests for physician-hastened death would be minimized.”^6^ The CSPCP advocates for harm reduction and argues that palliative medicine does not have a primary role in the implementation of PAD as identified in the SCC ruling.^6^

While PAD is becoming part of the medical landscape, there has been little commentary or study on the role that resident physicians will have within this new paradigm. Although the literature remains silent on the appropriate time at which to implement education on PAD within training programs, a focused educational strategy will become important now that legislation has opened the door to the practice of PAD. We conducted a survey study to determine British Columbia (BC) residents’ prior educational experiences in palliative medicine and PAD, residents’ attitudes towards the legalization of PAD, and how residents see PAD being implemented in the healthcare system. We explored how the responses of family and internal medicine residents compared to those of the collective resident population, since family and internal residents may be more likely to encounter PAD requests as part of their primary or acute care of medically complex and terminally ill patients. Our data may be useful in creating educational recommendations regarding palliative medicine and PAD for residency programs across Canada, and in guiding future research.

## Methods

### Design and procedure

The study is a mixed quantitative and qualitative cross-sectional survey-based study (see Appendix A). We obtained ethics approval through the University of British Columbia (UBC) Behavioural Research Ethics Board. The online survey opened to all resident physicians in BC on March 27^th^, 2016 and was closed on April 17^th^, 2016. We distributed the survey link to residents through their professional association, Resident Doctors of BC,^7^ and via their residency program. Responses were anonymous and participation was voluntary. We obtained written informed consent.

### Research materials

We created the survey using FluidSurveys™, an online platform available through UBC. The survey was composed of a variety of questions including: demographic information, close-ended questions, graded Likert scale questions, and a comments section. We designed the survey with the assistance of a professional survey designer to optimize internal and content validity. We distributed consent forms with the recruitment email (see Appendix B).

### Participants and study eligibility

**Figure 1:**
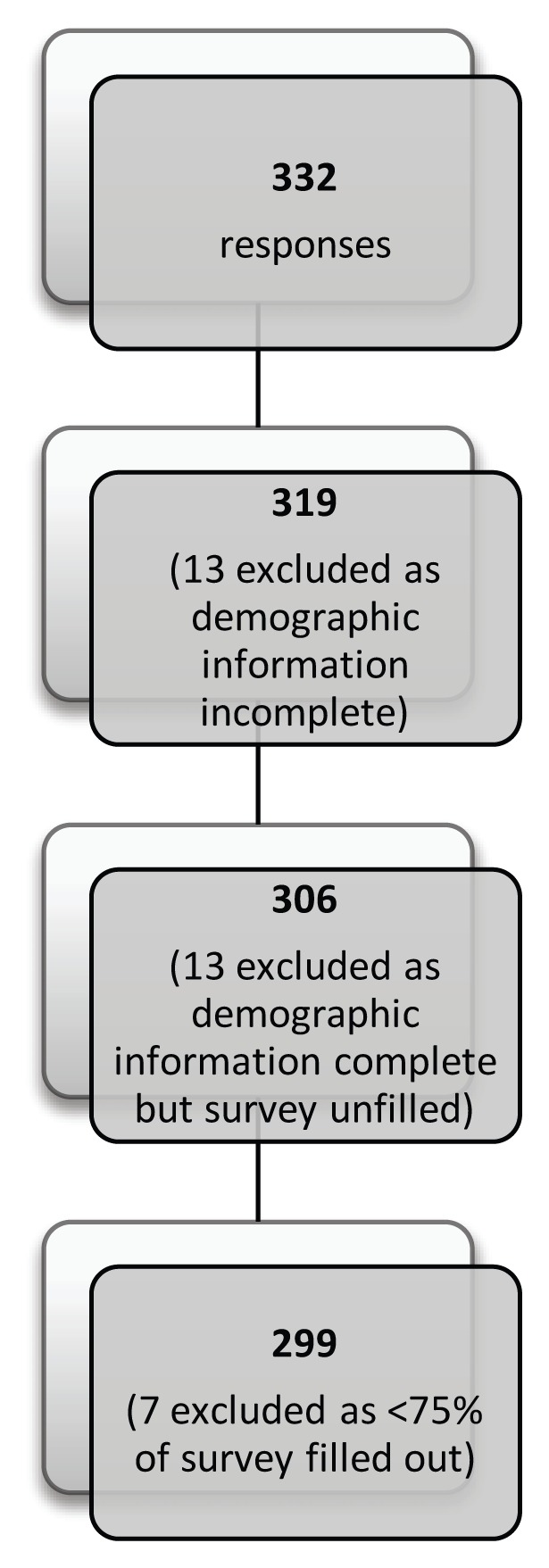
Eligibility and selection of respondent surveys for data analysis

The study population was the 1270 members of Resident Doctors of BC.^7^ For responses to be included in the data analysis, respondents were required to complete the demographic information, and at least 75% of the survey questions (Figure 1).

### Data analysis

We analyzed the data using Sigma Stat software. Family medicine and internal medicine resident responses were compared with Fisher’s exact test to all other resident responses and expressed using p-values ≤0.05 to explore statistically significant trends. We used a thematic analysis to analyze the qualitative comments section. One author reviewed the comments, coded responses into categories, and then identified overarching themes. A second author verified the coding into themes to ensure general agreement.

## Results

Two hundred and ninety-nine respondents met our eligibility criteria as outlined in Figure 1, and were included in the data analysis. This represents a 24% response rate, surpassing the 295 respondents required to attain a 95% CI for sufficient sampling of this participant group. Table 1 summarizes the demographic details of respondents. All residency programs at UBC were represented and of the 299 respondents, 64% were from family or internal medicine residency programs.

### Resident education in palliative care and physician assisted death

As outlined in Table 2 (Appendix C), less than half of all residents (44%, n=131) have received ≥5hrs of education in palliative care during their training, with family medicine residents significantly more likely (54% vs. 44%, p=0.026), and internal medicine residents significantly less likely (31% vs. 44%, p=0.027), compared to residents in other programs to have received ≥5hrs. Residents most commonly received palliative care education in the form of lectures (77%, n=230), core rotations (25%, n=76), and seminars (21%, n=63). Family medicine residents were far more likely to have completed a core palliative care rotation than residents in other programs (50% vs. 6%, p=0.0048).

The majority of overall respondents (72%, n=218) agreed (somewhat or strongly) that more education in palliative care should be part of their residency program, with internal medicine residents significantly more likely to agree (somewhat or strongly) compared to residents in other programs (90% vs. 72%, p=0.01).

Seventy-five percent (n=223) of overall respondents had no education about PAD and most residents (64%, n=192) disagreed (somewhat or strongly) that their residency programs provided sufficient education to make an informed decision about PAD, with no differences across training programs. The majority of residents (85%, n=255) agreed (somewhat or strongly) that residency programs should provide more education on PAD, regardless of the specific training program.

### The Supreme Court of Canada ruling and PAD implementation

Nearly all respondents (98%, n=293) were aware of the SCC ruling that legalized PAD and the majority (67%, n=199) were in agreement (somewhat or strongly) with the decision regardless of training program, as outlined in Table 2 (Appendix C). Based on the ruling, the majority of residents would help a competent, consenting dying patient end her/his life if requested, with 10% (n=29) in full agreement and 49% (n=146) in agreement presuming appropriate checks and balances were in place, with no difference across training programs.

A minority of residents (39%, n=116) agreed (somewhat or strongly) with being comfortable discussing the SCC ruling with patients, with family medicine residents significantly more likely to report being comfortable (49% vs. 31%, p=0.0006), and internal medicine residents significantly less likely (24% vs. 39% p=0.043) compared to residents in other programs. Amongst residents who were comfortable discussing the SCC ruling with patients, 68% (n=79) had zero hours of training on PAD in their residency programs.

Approximately half (47%, n=142) of all respondents thought that palliative physicians would be the ones to provide PAD. Residents outside of family practice were more likely to think that PAD would be provided by palliative medicine than were residents in family practice (53% vs. 41%, p=0.023). Family medicine respondents were significantly more likely to think that PAD would be provided by family physicians than were residents in other programs (20% vs. 6%, p=0.0003).

The majority of respondents (96%, n=283) agreed (somewhat or strongly) that physicians who provide PAD should receive additional formal training.

### Qualitative comments

Sixty-one of the respondents completed the comments section. Our qualitative analysis found three common themes that emerged from the responses:^1^ comments on the present appropriateness of residents participating in education on PAD,^2^ Specific concerns regarding PAD legislation,^3^ and personal ethical opinions towards PAD.

Comments from 19 respondents expressed a strong desire for more education in PAD. The following verbatim comments are representative of such views:

I think this is a very important topic for us to discuss. We certainly need to learn about this in our residency training.

PAD is a great stride forward for Canada and should be supported by the college through training.

As captured in the following statements, two respondents thought that the role of residents in the current legislation is still undefined and education for residents is currently not appropriate:

I think we do not know enough at this stage for teaching.

I think it's still too early to start training on something that has yet to really gain momentum or real-life application.

Comments from 14 respondents contained specific concerns regarding PAD legislation. Some of these statements were general comments about legislation such as the following:

I think that the appropriate checks and balances need to be in place before PAD is allowed.

Others comments questioned how the new legislation might specifically affect their patient population:

Many of my patients experience a single static traumatic event (i.e. Spinal Cord Injury) and the College recommendations are that the patient should receive PAD within 2 weeks of requesting it. The research shows that 90% of SCI patients wish they were dead soon after the injury, but only 10% feel the same way at one year. I'm concerned I won't be able to convey that hope to a competent patient with acute SCI.

Comments from 28 respondents expressed personal ethical opinions towards PAD. Statements were generally either strongly supportive or oppositional towards PAD. The following two statements capture the polarized views amongst residents:

I am heart broken to think that our specialty, which has brought life to people for so many years, is degrading itself to the point where physicians will be associated with ending precious lives.

I am pleased Canada is being so progressive with PAD and weight being put on patient dignity and autonomy.

## Interpretation

To our knowledge, this is the first survey to be conducted in Canada that evaluates resident perceptions regarding palliative care and PAD education. Our study demonstrates a heterogeneous educational exposure to palliative medicine in different training programs across BC, and a strong demand for more training in palliative care and PAD education in all residency programs. Family practice residents receive the most training in palliative care and report the greatest comfort in discussing PAD with patients, but persist in wanting more palliative education during residency. Internal medicine residents report having received the least education in palliative medicine and possess the least amount of comfort discussing PAD with patients, and perhaps consequently the strongest desire of any group for more palliative care education. One factor that may contribute to the observed educational deficit is that some of the respondents are at an early stage in their training; these residents may receive further education in palliative medicine and PAD by the time they complete their training.

Despite the general gap in PAD education across all programs, resident approval of the SCC ruling is generally similar to that of practicing physicians, with the exception that residents are possibly more willing to “help a competent, consenting dying patient end her/his life if requested.” In the 2015 CFPC ePanel, a slight majority of family physicians agreed with the SCC ruling and would consider providing PAD to patients, similar to the agreement levels shown by residents surveyed in this study.^5^ However, it is interesting to note a significant difference between resident respondents who stated they would consider providing PAD in this survey (59%), and that of the 2014 and 2015 CMA general member polls, where only 27% and 29% of practicing physicians from all disciplines would consider providing PAD.^4^,^8^ There are multiple hypotheses to explain the difference in opinion between residents and senior physicians, including medical, cultural, and experiential differences. Practicing physicians with greater experience caring for terminally ill patients are less likely to support PAD.^9^ Conversely, younger respondents demonstrate stronger support for legalizing euthanasia compared with older respondents.^9^–^13^ In addition, a generational paradigm shift should be considered, taking into account multiple American surveys^9^–^14^ showing a decade-by-decade rise in physician and public approval rates for physician assisted suicide. Given the younger age of residents versus practicing physicians, this generational shift could contribute to the observed discrepancy in approval for PAD. However, because 86% of the respondents fell within the 26–35 age range and no survey question addressed the association between age and PAD approval, our study cannot adequately assess the relationship observed between age and PAD approval noted in other studies.

In this study, 59% of resident respondents agreed that they would “help a competent, consenting dying patient end her/his life if requested.” However, difficulty arises when assessing what this means to an individual resident. Helping provide PAD can encompass discussion of goals of care and alternative medical treatments, referral to a specialist in PAD, or the injection or supply of lethal medications. This distinction is important because some residents in this study may have interpreted “helping” a patient to end their life as a referral to a specialist in PAD, rather than directly providing PAD itself. This is supported by the observation that almost half of residents surveyed assumed that PAD will be provided by palliative physicians, and may therefore believe their own role would be peripheral. If residents were asked to directly provide PAD it is possible their agreement level would diminish, a question that could be explored further in future research.

The residents’ assumption that palliative care physicians will be a main provider of PAD is contradicted by the position of The Canadian Society of Palliative Care Physicians (CSPCP), which distinctly separates PAD provision and the therapeutic role of palliative medicine.^6^ Further, many individual palliative care physicians are strongly opposed to participation in PAD. The conflation of PAD and palliative medicine appears a common misconception, perhaps because both areas deal with medical care at the end of life. However, the CSPCP is clear to establish that the preferred focus of palliative medicine is symptom management and not the purposeful hastening of death, the specific goal in PAD. It is therefore important that resident education explores how and by whom PAD will be provided. This may be subject to local variation as each provincial college and health care institution interprets and institutes a PAD delivery structure. Interestingly, family medicine residents, who report the most exposure to palliative medicine teaching and for whom palliative care education is an accreditation standard, are less likely to believe that palliative care specialists will provide PAD. This may indicate that education in palliative care provides residents more insight into the preferred focus of palliative care.

Because the changes to Canadian laws governing PAD are very recent, it is not surprising that most residents report limited training in this topic. However, despite this reported educational gap, it is notable that approximately one-third of residents feel comfortable discussing the SCC ruling and PAD with their patients and two-thirds would consider providing PAD to their patients presuming adequate regulation. Residents may be taking the initiative to educate themselves by following discussions supported by the CMA or local provincial colleges. However, a lack of education in this evolving area may lead to an increased risk of false assumptions about the application of PAD, which in turn could cause substantial and irreversible errors in patient care. Immediately following the February 2015 SCC ruling, researchers discovered major misunderstandings regarding PAD amongst practicing physicians in Quebec.^15^ Formal educational opportunities related to PAD may clarify misconceptions and could ultimately help residents to form stronger therapeutic relationships with their patients who are grappling with the option of PAD. When medical residents receive formal education in end of life care, their perceived comfort level and attitudes towards providing palliative care improve.^16^,^17^

Through the comments section in the survey, resident respondents advocated for learning opportunities surrounding PAD in the form of standardized case studies, lectures, and workshops. Additionally, the opportunity to discuss PAD uncovers strong ethical viewpoints, as seen in the comments, and education around PAD could also serve to facilitate a deeper understanding and respect of the professional and ethical uncertainties felt towards this subject. Presently there is no evidence or guideline dictating the quantity or type of education sufficient to ensure general competency in the area of PAD, and future research will be required to explore this important question. Many residents also expressed an interest in having more access to palliative care training through electives and formal teaching. Increased exposure to palliative medicine will be important in developing the skills necessary to care for patients at the end of their life, but also to help reduce the misconception that palliative medicine will have a primary role in PAD at the present time.

Our study received responses from every residency training program in BC, but the survey results may not have generalizability for smaller training programs. Sixty-five percent of respondents were from family medicine and internal medicine, although these residents only account for 40% of the total resident population in BC. For example, only 2% (n=7) of responses received were from neurology residents, a group that may experience a disproportionate number of PAD requests in the context of neurodegenerative diseases.^18^ Despite having third parties distribute the survey, a pre-screening bias is possible because family and internal medicine residents were involved in the running of the study. Moreover, there may be a self-selection bias such that residents who did not believe that PAD was relevant to their specialty chose not to participate in the study. We created and distributed the survey after the Supreme Court had ruled in favour of legalizing PAD, but prior to the provincial colleges establishing much of the regulatory mechanisms for PAD following the passing of Bill C-14.^3^ The evolving nature of PAD regulation may therefore have impacted residents’ understanding of the issue and their ensuing survey responses.

## Conclusion

Residents receive little education about palliative care and PAD, and want more robust education in these areas. Residents have significant misunderstandings regarding the interactions between palliative care and the provision of PAD, and education in these areas should be provided in all residency training programs in order to meet the needs of future practicing physicians. Further research could explore what types and what quantity of educational opportunities are sufficient to meet resident needs. The regulation and practice of PAD is rapidly evolving, and future research should explore if opinions and experiences with respect to PAD change over time, and how the opinions of residents in different programs and levels of training compare on these important questions.

## Figures and Tables

**Table 1:** Baseline characteristics of study participants

Characteristic	Study participants (n = 299)
Gender	
Male	127 (42.1)
Female	172 (57.9)

Age	
>40	11 (3.7)
36–40	18 (6.0)
31–35	76 (25.4)
26–30	182 (60.9)
20–25	12 (4.0)
<20	0

Residency program	
Family Medicine	133 (44.5)
Internal Medicine	61 (20.4)
Psychiatry	30 (10.0)
Surgical Program*	23 (7.7)
Anesthesia	15 (5.0)
Emergency Medicine	13 (4.3)
Pediatrics	8 (2.7)
Other†	16 (5.4)

Year of training	
PGY1	109 (36.5)
PGY2	113 (37.8)
PGY3	43 (14.4)
PGY4	16 (5.4)
PGY5	13 (4.3)
PGY6	4 (1.3)
PGY7 or higher	1 (0.3)

* General surgery, neurosurgery, obstetrics & gynecology, ophthalmology, orthopedics, plastic surgery, urology

† Dermatology, radiology, medical microbiology, neurology, radiation oncology, physiatry/rehab medicine, public health PGY denotes post graduate year of study

## Footnotes

* Since this study’s inception, the federal government passed Bill C-14 and there have been many developments in the regulation and implementation of PAD. The term Medical Assistance in Dying (MAiD) is now the accepted terminology used to reflect the different modalities available to patients (euthanasia and assisted suicide) and the fact that practitioners other than physicians can be involved in the process. However, we use PAD throughout the body of our paper as it reflects the language used in our study materials and was the preferred term at the time of data collection.

## Appendix A Online survey

Evaluation of BC resident education on physician assisted death (supported by FluidSurveys™)

### DEMOGRAPHIC INFORMATION

1. Demographic information
  - Male/Female/Other
  - Age ranges: <20, 20–25, 26–30, 31–35, 36–40, >40
  - Program: Drop down list of all residency programs in BC
  - Year in residency program: Drop down list of R1, R2, R3, R4, R5, R6, R7+

### EDUCATION

2. How much palliative care education have you had since beginning your residency training?
  - 0 hours
  - 1–4 hours
  - 5+ hours
3. My education in palliative care in residency has consisted of (click all that apply):
  - Lectures
  - Core rotation
  - Elective rotation
  - Seminar
  - Conference
  - I have had no training in palliative medicine
  - Other
4. More education on palliative care should be a part of my residency program.
  - Likert scale
5. How much education on physician assisted death have you had since beginning your residency training?
  - 0 hours
  - 1–4 hours
  - 5+ hours
6. My residency program provides me with education to make an informed decision around physician assisted death.
  - Likert scale
7. More education on PAD should be a part of my residency program.
  - Likert scale

### AWARENESS OF LEGISLATION AND UNDERSTANDING OF LEGISLATION

8. Are you aware that the Supreme Court of Canada decriminalized physician assisted death (Carter v Canada Feb 2015)?
  - Yes
  - No - If so, skip to question 10
9. Do you agree with the recent Supreme Court of Canada decision that struck down sections of the Criminal Code that prohibit physicians from helping patients die?
  - Yes
  - No
  - I don’t know enough about the Supreme Court of Canada’s decision to answer
  - I prefer not to answer
10. A 72 year old female diagnosed with early dementia, with a clear understanding of the trajectory of her disease, requests PAD. At this time, she is functional in her activities of daily living, and instrumental activities of daily living. There are no depressive features.
  - I think PAD is appropriate for this patient.
  - No, I don’t think PAD is appropriate for this patient.
  - I don’t have enough education on PAD to make an informed decision in this situation.
11. A 60 year old male with ALS is on continuous BiPAP. He is beginning to have difficulties with swallowing but has stated that he does not want to have a feeding tube. He still has some function of one arm and has requested PAD.
  - I think PAD is appropriate for this patient.
  - No, I don’t think PAD is appropriate for this patient.
  - I don’t have enough education on PAD to make an informed decision in this situation.
12. A 45 year old male is recently diagnosed with advanced pancreatic cancer. He has refused surgery or chemotherapy. His ambulation is reduced, he is unable to participate in his hobbies and he occasionally requires assistance with self-care. He is also exhibiting signs of depressive features.
  - I think PAD is appropriate for this patient.
  - No, I don’t think PAD is appropriate for this patient.
  - I don’t have enough education on PAD to make an informed decision in this situation.
13. Would you help a competent, consenting dying patient end her/his life if requested?
  - Yes – it is the patient’s choice
  - Never
  - Yes – if the appropriate and rigorous checks and balances are in place
  - I don’t have enough education on PAD to make an informed decision at this time.

### IMPLEMENTATION

14. I am comfortable discussing the Supreme Court ruling with patients.
  - Likert scale
15. I am comfortable discussing PAD with patients.
  - Likert scale
16. I think PAD will be provided by (choose one):
  - Geriatric medicine
  - Palliative medicine
  - Anaesthesia
  - Family medicine
  - Internal medicine
  - Psychiatry
  - Other
  - Any provider
17. Physicians who provide PAD should require additional formal training.
  - Likert scale

### COMMENTS (2 boxes with following headings)

1. How do you think your gaps in knowledge for palliative care and PAD would be best addressed by your residency training program and other organizations?
2. General Comments

Likert-scale:

- Strongly agree
- Somewhat agree
- Neither agree or disagree
- Somewhat disagree
- Strongly disagree

## Appendix B Consent form

Vancouver Fraser Site
  RCH Medical Education
  330 E Columbia Street
  New Westminster, BC, V3L 3W7

Consent Form

Family Medicine Research Project:

Evaluation of BC Resident education on physician assisted death

### 1. STUDY TEAM

Principal investigator:

Dr. Charlie Chen
  Hospice Palliative Care Consultant
  Royal Columbian Hospital & New Westminster, BC
  Voicemail 604-520-4007
  Fax 604-800-1651

Co-investigators:

Dr. Sonia Paul
  Resident, Vancouver Fraser Site
  UBC Faculty of Medicine, Department of Family Medicine
  tspaul@alumni.ubc.ca
  778-322-9534
Dr. Tom (Shuan Ta) Tang
  Resident Vancouver Fraser Site
  UBC Faculty of Medicine, Department of Family Medicine
  tomtang@alumni.ubc.ca
  604-839-3832
Dr. Jocelyn Chase
  Internal Medicine, Geriatrics
  Providence Health
  jchase@providencehealth.bc.ca
Dr. David Spicer
  Resident, Internal Medicine
  UBC Faculty of Medicine, Department of Internal Medicine
  davidaspicer@alumni.ubc.ca
  778-233-2647

### 2. WHY ARE WE DOING THIS STUDY?

The Canadian Supreme Court has ruled that physician assisted death (PAD) is no longer a criminal offense. We are inviting you to take part in this study because you are a resident physician training in BC. The goal is to learn more about what you know of this ruling and if you think more education is needed on this topic.

### 3. HOW IS THIS STUDY DONE?

Participation in the study is voluntary. You can refuse to participate or withdraw from the study at any point without penalty. If you agree to participate you will be asked to answer some questions. We will ask you about your level of training in end of life care as well as your opinion on physician assisted death. We will also ask you about your opinion on scenarios of physician assisted death.

The survey is done online and will take 5–15 minutes to complete. This online survey is done through FluidSurveys. Survey responses are hosted by a web survey company located in Canada and complies to the BC Freedom of Information and Protection of Privacy Act. This survey does not ask for personal identifiers or any information that may be used to identify you. The web survey company servers record incoming IP addresses of the computer that you use to access the survey but no connection is made between your data and your computer’s IP address. If you choose to participate in the survey, you understand that your responses to the survey questions will be stored and accessed in Canada only. The security and privacy policy for the web survey company can be found at the following link: https://fluidsurveys.com/about/privacy

The responses will be compiled in an anonymous format and analyzed to look for trends. The results of this study will be presented at the UBC Family Medicine Program Resident Research Day in June 2016. If appropriate, they may also be published in a future publication. Any alternative use of the data will be subject to review by the Research Ethics Board.

### 4. IS THERE ANY WAY BEING IN THIS STUDY COULD BE BAD FOR YOU?

We do not think there is anything in this study that could harm you or be bad for you. Some of the questions we ask might upset you or seem sensitive. Please let one of the study staff know if you have concerns. You do not have to complete the survey if you do not feel comfortable answering any of the questions.

If you have any concerns or complaints about your rights as a research participant and/or your experience during the study, you can contact the Research Participant Complaint Line in the UBC Office of Research Ethics at 604-822-8598 or at RSIL@ors.ubc.ca.

### 5. WHAT ARE THE BENEFITS OF PARTICIPATING?

You may help promote change in your residency training to include more end-of-life and palliative care. Other future residents may also benefit from what we learn in this study. The study may help you reflect on your attitudes towards PAD.

### 6. HOW WILL YOUR IDENTITY BE PROTECTED?

Your confidentiality will be respected. Information that discloses your identity will not be released without your consent unless required by law. All data records will be kept either online through FluidSurveys or on a computer hard disk. The online data is only accessible to the study team through a password-protected login. Data that is downloaded onto a computer hard drive for analysis will be stored in password protected files. The computer will also be password protected. The results of our research will not include any identifiers.

### 7. DO YOU RECEIVE ANYTHING FOR PARTICIPATING?

When you complete the survey, you will be entered into a draw to win one of four Starbucks gift cards, each valued at $25.

### 8. WHO CAN YOU CONTACT IF YOU HAVE QUESTIONS ABOUT THE STUDY?

If you have questions or concerns about what we are asking of you, please contact one of the study staff. The names and telephone numbers are listed at the top of this form.

### 9. PARTICIPANT CONSENT AND SIGNATURE PAGE

Taking part in this study is voluntary. You have the right to refuse to participate. If however you decide to take part, you may choose to pull out of the study at any time without giving a reason and without any consequence.

Your participation in the survey implies consent to the study as described above.

## Appendix C

**Table 2:** Survey responses from all residents by training program

Question	All Residents	Family practice residents	Internal medicine residents
n =	299	133	61

**How much palliative care education have you had since beginning your residency training?**			

0 hours	47 (16)	19 (14)	7 (11)

1–4 hours	110 (37)	38 (29)	33 (54)

5 + hours	131 (44)	72 (54)	19 (31)
No response given	11 (3)	4 (3)	2 (4)

**More education on palliative care should be apart of my residency program**			

Strongly disagree	7 (2)	5 (4)	0

Somewhat disagree	15 (5)	9 (7)	0

Neutral	58 (19)	37 (28)	5 (9)

Somewhat agree	115 (39)	39 (29)	25 (41)

Strongly agree	103 (35)	43 (32)	30 (49)
No response given	1 (0)	0 (0)	1 (1)

**My education in palliative care in residency has consisted of (click all that apply)**			

Lectures	230 (77)	100 (75)	52 (85)

Core rotation	76 (25)	66 (49)	3 (5)

Elective rotation	48 (16)	16 (12)	6 (10)

Seminar	63 (21)	18 (14)	19 (31)

Conference	25 (8)	12 (9)	2 (3)

I have had no training in palliative medicine	37 (12)	14 (11)	7 (11)

Other	31 (10)	13 (10)	5 (8)

**How much education on PAD have you had since beginning your residency training?**			

0 hours	223 (75)	106 (80)	45 (74)

1–4 hours	69 (23)	26 (19)	14 (23)

5 + hours	7 (2)	1 (1)	2 (3)

**More education on PAD should be a part of my residency program**			

Strongly disagree	8 (3)	2 (2)	0

Somewhat disagree	4 (1)	2 (2)	0

Neutral	32 (11)	12 (9)	6 (10)

Somewhat agree	137 (46)	68 (51)	23 (38)

Strongly agree	118 (39)	49 (36)	32 (52)

**My residency program provides me with education to make an informed decision around PAD**			

Strongly disagree	105 (35)	35 (26)	24 (39)

Somewhat disagree	87 (29)	45 (34)	20 (33)

Neutral	60 (20)	29 (22)	12 (19)

Somewhat agree	37 (13)	21 (16)	4 (7)

Strongly agree	10 (3)	3 (2)	1 (2)

**Are you aware that the SCC decriminalized physician assisted death (Carter v Canada Feb 2015)?**			

Yes	293 (98)	129 (97)	60 (98)

No	6 (2)	4 (3)	1 (2)

**Do you agree with the recent SCC decision that struck down sections of the Criminal Code that prohibit physicians from helping patients die?**			

Yes	199 (66)	91 (68)	43 (70)

No	41 (14)	18 (14)	6 (10)

I do not know enough about the SCC ruling to answer	51 (17)	20 (15)	11 (18)

No response given	8 (3)	2 (3)	1 (2)

**Would you help a competent, consenting dying patient end her/his life if requested?**			

Yes, it is the patient's choice	29 (10)	12 (9)	5 (8)

Yes, if appropriate checks and balances are in place	146 (49)	67 (50)	29 (48)

Never	49 (16)	18 (14)	13 (21)

I do not have enough education on PAD to make a decision at this time	71 (24)	35 (26)	13 (21)
No response given	4 (1)	1 (1)	1 (2)

**I am comfortable discussing the SCC ruling with patients**			

Strongly disagree	52 (17)	17 (13)	15 (25)

Somewhat disagree	80 (27)	32 (24)	19 (31)

Neutral	51 (17)	19 (14)	11 (18)

Somewhat agree	89 (30)	54 (41)	11 (18)

Strongly agree	27 (9)	11 (8)	5 (8)

**I am comfortable discussing PAD with patients**			

Strongly disagree	62 (21)	20 (15)	23 (38)

Somewhat disagree	101 (34)	44 (33)	18 (30)

Neutral	45 (15)	17 (13)	8 (13)

Somewhat agree	70 (23)	43 (32)	10 (16)

Strongly agree	21 (7)	9 (7)	2 (3)

**I think PAD will be provided by**			

Palliative Medicine	142 (47)	54 (41)	31 (51)

Any Provider	83 (28)	38 (29)	20 (32)

Family Medicine	36 (12)	26 (20)	4 (7)

Other	23 (8)	7 (5)	6 (10)

Anesthesia	7 (2)	3 (2)	0
No response given	8 (3)	5 (3)	0 (0)

**Physicians who provide PAD should require additional formal training.**			

Strongly disagree	0	0	0

Somewhat disagree	4 (1)	1 (1)	0

Neutral	9 (3)	6 (5)	2 (3)

Somewhat agree	69 (23)	35 (26)	15 (25)

Strongly agree	217 (73)	91 (68)	44 (72)

Notes:

SCC denotes Supreme Court of Canada

PAD denotes Physician Assisted Death
